# Supplementary material for: An enhanced transcription factor repressilator that buffers stochasticity and entrains to an erratic external circadian signal
Source: Front Syst Biol. 2023 Dec 13;3:1276734. doi: 10.3389/fsysb.2023.1276734 (PMC12342007; doi:10.3389/fsysb.2023.1276734)
Supplement: Supplementary file 1 [file Image1.pdf]

SUPPLEMENTARY FIGURES

Figures 1–3 show the dynamics of the systems for a particular pattern of the stochastic external light signal. Figures S1–S3 show dynamics for the same systems with different realizations of the stochastic external input. The figure labeling associates Fig. X with Fig. SX, for $X = 1, 2, 3$, so that pairs describe the same system but with different stochastic inputs. Comparing two different runs of the same system helps to visualize how different stochastic inputs alter system dynamics.

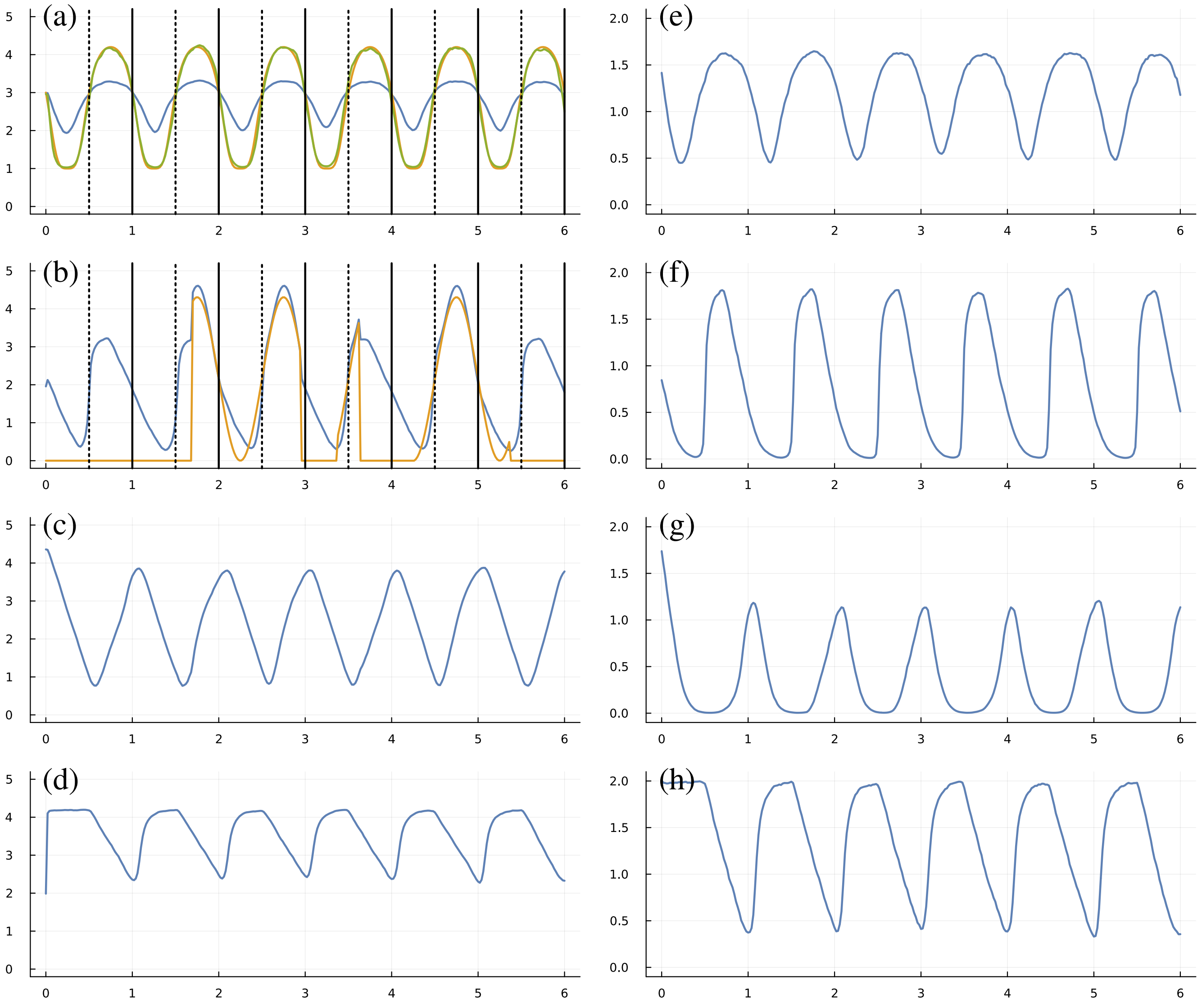

Figure S1. Another plot of the circadian dynamics of the TF network from run sde–4_1_t4, illustrating the consequences of a different pattern for the stochastic external light signal.

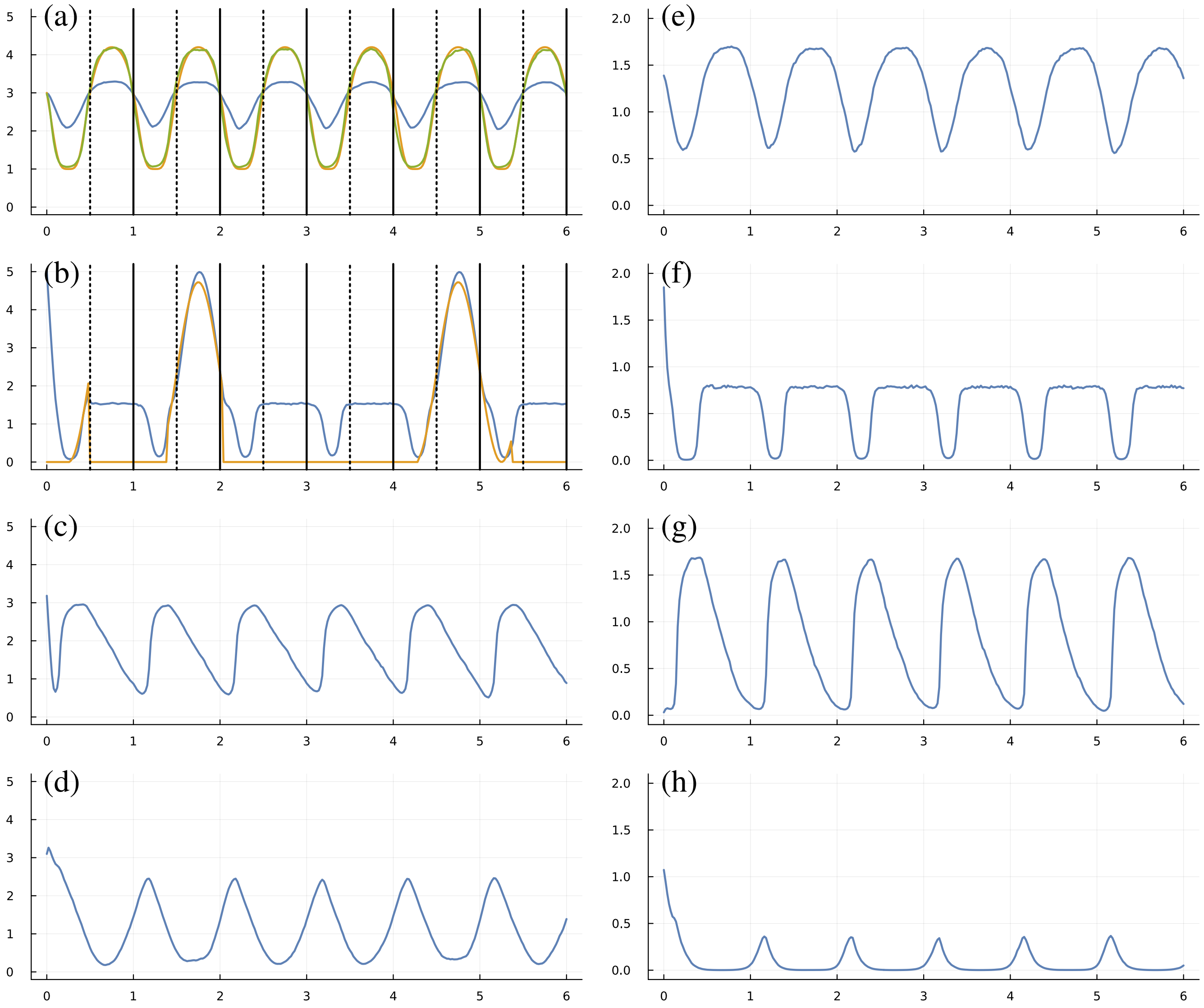

Figure S2. Another plot of the circadian dynamics of the TF network from run sde–4_2_t4, illustrating the consequences of a different pattern for the stochastic external light signal.

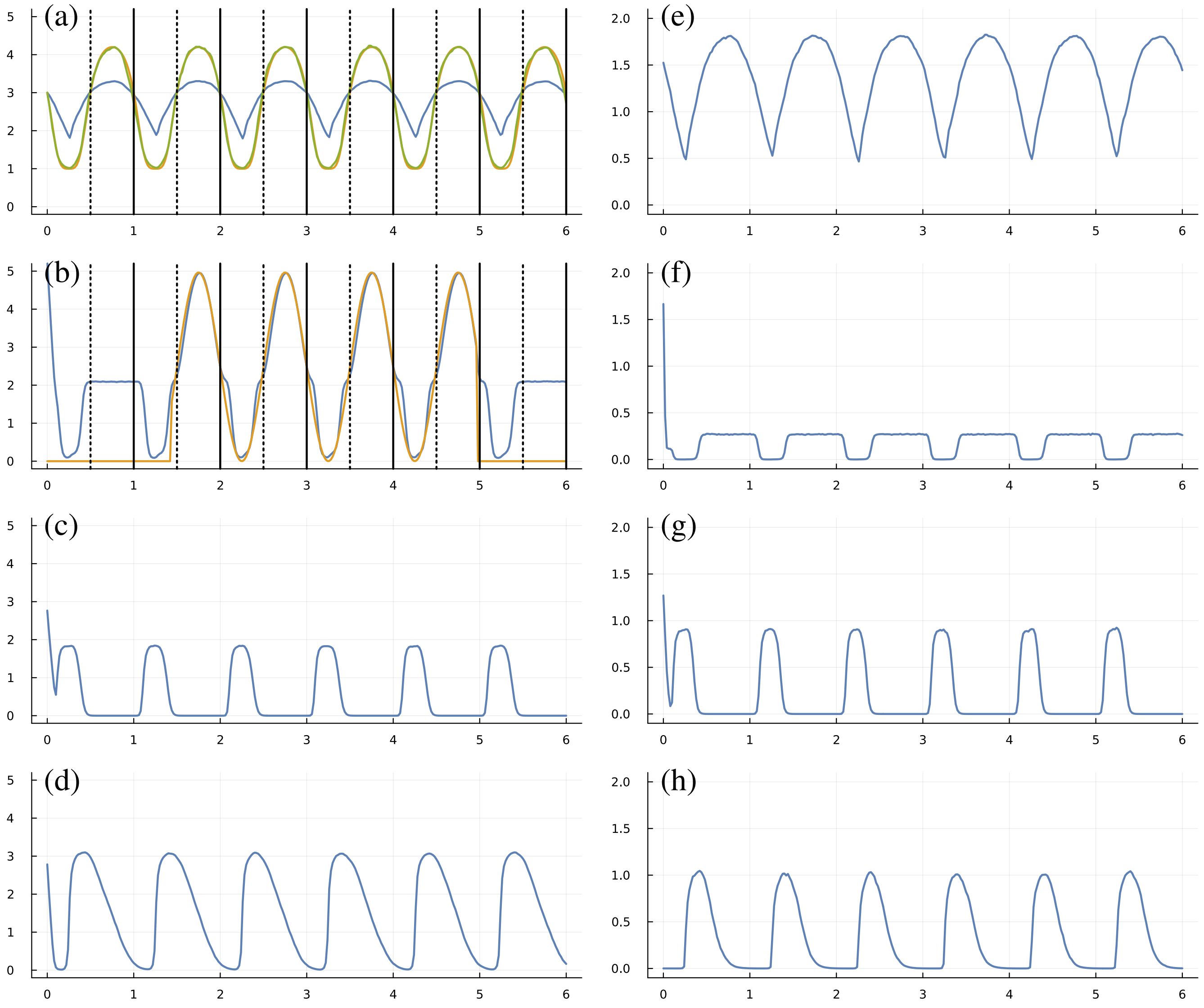

Figure S3. Another plot of the circadian dynamics of the TF network from run sde–4_8_t4, illustrating the consequences of a different pattern for the stochastic external light signal.
